# Altered structural connectivity networks in dementia with lewy bodies

**DOI:** 10.1007/s11682-020-00444-x

**Published:** 2021-01-29

**Authors:** Nicolas Nicastro, Elijah Mak, Ajenthan Surendranathan, Timothy Rittman, James B. Rowe, John T. O’Brien

**Affiliations:** 1 Department of Psychiatry, University of Cambridge, Cambridge, UK; 2 Division of Neurology, Department of Clinical Neurosciences, Geneva University Hospitals, 4, rue G. Perret-Gentil, 1205 Geneva, Switzerland; 3 Department of Clinical Neurosciences, University of Cambridge, Cambridge, UK; 4 Medical Research Council Cognition and Brain Sciences Unit, Cambridge, UK

**Keywords:** Dementia, Graph theory, Brain network, Cortical thickness, MRI

## Abstract

The impairment of large-scale brain networks has been observed in dementia with Lewy bodies (DLB) using functional connectivity, but the potential for an analogous effect on structural covariance patterns has not been determined. Twenty-four probable DLB subjects (mean age 74.3 ± 6.7 years, 16.7% female) and 23 similarly aged Controls were included. All participants underwent 3T MRI imaging with high-resolution T1-weighted magnetization-prepared rapid gradient echo (MPRAGE) sequence. Graph theoretical analyses were performed using variation in regional cortical thickness to construct a structural association matrix with pairwise Pearson correlations. Global and nodal graph parameters were computed to assess between-group differences and community structure was studied in order to quantify large-scale brain networks in both groups. In comparison to Controls, DLB subjects had decreased global efficiency, clustering, modularity and small-worldness of structural networks (all p < 0.05). Nodal measures showed that DLB subjects also had decreased clustering in bilateral temporal regions and decreased closeness centrality in extensive areas including right middle frontal, left cingulate and bilateral occipital lobe (all false-discovery rate (FDR)-corrected q < 0.05). Whereas four distinct modules could be clearly identified in Controls, DLB showed extensively disorganized modules, including default-mode network and dorsal attentional network. Our results suggest a marked impairment in large-scale brain structural networks in DLB, mirroring functional connectivity networks disruption.

## Introduction

Dementia with Lewy bodies (DLB) is the second-leading degenerative dementia in older people after Alzheimer’s disease (AD), accounting for 10–15% of cases (Jellinger and Attems 2011; Arnaoutoglou et al. 2019). It is clinically characterized by core features including recurrent visual hallucinations, rapid eye movement (REM) sleep behaviour disorder (RBD), cognitive fluctuations and parkinsonism (McKeith et al. 2017). Alpha-synuclein protein deposition with a variable degree of AD co-pathology represents the neuropathological hallmark of DLB (Spillantini et al. 1997; Gomperts 2016). Structural MRI surface-based morphometry has shown cortical thinning in posterior regions, especially in cingulate and parietal regions, with a relative preservation of medio-temporal lobe compared to AD (Watson et al. 2015; van der Zande et al. 2018). In addition to molecular and structural brain changes, functional connectivity studies with resting-state functional MRI (fMRI) applying either seed-based or independent component analysis have revealed distinct network disruptions, but sometimes with contrasting results. In fact, increased, reduced or even preserved default-mode network (DMN) connectivity has been described in DLB (Galvin et al. 2011; Lowther et al. 2014; Schumacher et al. 2018). Similarly, reports on other large-scale networks are heterogeneous, with impaired connectivity being described for salience, executive (Lowther et al. 2014), attentional (Kobeleva et al. 2017), basal ganglia (Lowther et al. 2014), fronto-parietal (Peraza et al. 2014) and sensori-motor networks (Lowther et al. 2014; Peraza et al. 2014; Schumacher et al. 2018). Interestingly, only one study described impaired visual network disruption (Sourty et al. 2016), which can be surprising when we consider the prominent visuo-spatial impairment observed in DLB.

In addition, impaired metabolic connectivity networks based on ^18^F-FDG PET imaging has been described in DLB, e.g. in primary visual cortex, posterior DMN and dorsal attentional network (DAN) (Sala et al. 2019), occipital lobe, cerebellum and thalamus (Caminiti et al. 2017). Cognitive impairment negatively covaried bilateral parietal and left precuneus metabolism in DLB (Morbelli et al. 2019).

Graph theory provides a useful framework to assess the relationship between brain regions and their organization into large-scale networks (Bullmore and Sporns 2009). In fact, both structural and functional brain systems have features of complex networks, including small-world topology and clustering, which can be assessed with structural MRI, diffusion tensor imaging or electroencephalography (EEG) (Achard and Bullmore 2007; Gong et al. 2009). Brain graphs can be constructed to study the nervous system as a set of nodes (anatomical brain regions) and interconnecting edges (i.e. structural/functional connections) (Bullmore and Bassett 2011).

Graph theoretical analyses of cortical thickness in AD and mild cognitive impairment (MCI) have shown altered global and nodal networks, especially in temporoparietal regions (He et al. 2008; Pereira et al. 2016). However, it is unknown whether similar large-scale network disruptions are observed in DLB.

With the present case-controlled study, we aimed to assess structural covariance using graph theoretical analyses of variations in cortical thickness, in a cross-sectional case-controlled cohort of clinically probable DLB subjects. Based on functional connectivity findings and graph theoretical analyses reported in AD, our hypothesis was that DLB participants would exhibit large-scale network disruptions and regional impairment affecting posterior brain regions.

## Methods

### Participants

The present study was conducted within the Neuroimaging of Inflammation in MemoRy and Other Disorders (NIMROD) study (Bevan-Jones et al. 2017). We included 24 participants with probable DLB according to 2017 consensus criteria (McKeith et al. 2017). Subjects were aged > 50 years and had at least two-year clinical follow-up to confirm clinical progression and no diagnostic change. We also included 23 similarly aged healthy Controls, with mini-mental state evaluation (MMSE) scores greater than 26, absence of regular memory complaints, and without any significant medical illness. DLB clinical core features were collected: parkinsonism was assessed with Movement Disorder Society Unified Parkinson’s Disease Rating Scale (MDS-UPDRS) part III (Goetz et al. 2008), cognitive fluctuations with the Clinician Assessment of Fluctuation (CAF) scale (Walker et al. 2000), while the presence of visual hallucinations and RBD was assessed with history taking.

Patients were identified from the Memory clinic at the Cambridge University Hospitals NHS Trust, other local memory clinics, and from the Dementias and Neurodegenerative Diseases Research Network (DeNDRoN) volunteer registers. Healthy controls were recruited via DeNDRoN as well as from partners of participants. Informed written consent was obtained in accordance with the Declaration of Helsinki. The study was approved by the East of England Ethics Committee (Cambridge Central Research, Ref. 13/EE/0104).

### MRI acquisition

Participants underwent MRI imaging acquired at the Wolfson Brain Imaging Centre (University of Cambridge) on a 3T Siemens Magnetom Tim Trio scanner. A T1-weighted magnetization-prepared rapid gradient echo (MPRAGE) sequence was acquired with the following parameters: relaxation time (TR) = 2300 ms, echo time (TE) = 2.98 ms, field of view = 240 × 256 mm^2^, 176 slices, flip angle = 9°, isotropic 1mm^3^ voxel.

### Image preprocessing

The T1-MPRAGE images were processed with FreeSurfer v6 to obtain cortical thickness measurements in 34 regions of interest (ROIs) per hemisphere, based on the Desikan-Killiany parcellation scheme (Desikan et al. 2006). Briefly, for each MRI, the pial and white matter surfaces were generated and the cortical thickness was measured as the distance between their respective boundaries. Visual inspection was carried out blinded to group diagnosis and corrections were performed where necessary to ensure accurate skull stripping and reconstruction of white matter and pial surfaces.

### Network construction and analysis

For each of the 68 cortical ROIs, we performed linear regressions of cortical thickness to obtain residuals adjusting for age, sex, years of education and mean cortical thickness (Mak et al. 2016). These residuals were used to construct the brain graph, where every node corresponds to an anatomical brain region and the edges represent the correlation between them. For each diagnostic group, we built a 68 × 68 association matrix for which every entry was defined as the Pearson correlation (binary undirected graph) between each pair of ROI cortical thickness data. We used network densities (D) from 9–24% (in steps of 1%). These values were chosen as for D < 9%, the number of edges was inferior to the number of nodes, leading to a disconnected graph, whereas for D > 24%, the network was similar to random graphs and showed small-world index lower than 1.5. In order to assess between-group differences in network topology, we calculated the global and nodal parameters reflecting the various aspects of a brain graph, i.e. integration, segregation and centrality. The following global parameters were computed: local efficiency, clustering coefficient, modularity and small-worldness. The *local efficiency* is the average inverse shortest path length and is a measure of integration. The *clustering coefficient* assesses the presence of clusters in a graph. For each node, this can be calculated as the fraction of the node’s neighbours which are also neighbours of each other (Watts and Strogatz 1998). Thus, the clustering coefficient for each node can be averaged into the mean (global) clustering coefficient and represents a measure of segregation. The *modularity* represents the extent to which a network can be segregated into clearly delineated communities, reflecting a high number of edges within communities and lower number of edges between the different communities (Girvan and Newman 2002). The *small-world index* is a ratio between the characteristic path length and the global clustering coefficient. A small-world network features short paths but also high clustering coefficient, resulting in an optimal network architecture. As observed, several of the above measures are directly based on the evaluation of the characteristic path length. However, we did not report between-group results of this global measure. In fact, when testing integrative measures in a group of subjects with neurodegenerative conditions, it is considered as more suitable to use efficiency rather than characteristic path length in the analysis of disconnected networks (Rubinov and Sporns 2010).

To assess between-group differences in nodal network topology, we calculated nodal efficiency, nodal clustering and closeness centrality. *Nodal efficiency* represents the local average of the inverse shortest path length, whereas *nodal clustering* is the regional counterpart of global clustering coefficient, reflecting the ability of a network to segregate into specialized clusters in order to process a specific information (Rubinov and Sporns 2010). Finally, *closeness centrality* is defined as the inverse of the average shortest path length from one given node to all other nodes in the graph. This measure represents a useful way to assess whether a node is a brain hub, i.e. its centrality (van den Heuvel and Sporns 2013). In order to assess the large-scale brain modules in Controls and DLB, community structure was calculated using the Louvain algorithm at D = 15%, which is an average D value among the range of D used for the present study (9–24%) (Fox et al. 2005).

### Statistical analyses

Demographic data were analyzed with Stata software Version 14.2 (College Station, TX). Assessment of distribution for continuous variables was performed with Shapiro–Wilk test and visualization of histogram plots, followed by *t test* or *Mann–Whitney U test*, accordingly. Categorical variables were compared with Chi-Square test. Statistical significance was considered when p < 0.05.

All graph theoretical analyses were performed with BRain Analysis using graPH theory (BRAPH) software Version 1.0 (Mijalkov et al. 2017), a freeware running on MATLAB 2018b (version 9.5, Mathworks Inc, Sherborn, MA, USA). We thus built connectivity matrices in order to calculate global and nodal brain graph measures, in addition to performing non-parametric permutations (n = 5’000) for group comparisons and assess the different modules in both diagnostic groups. Significance threshold was set at two-tailed p < 0.05 for global measures and two-tailed false-discovery rate (FDR)-corrected q < 0.05 for nodal measures to correct for multiple comparisons. Global and nodal parameters in DLB were also compared to random graphs in order to ensure that significant findings against Controls were consistent. Estimation of modular similarities between Controls and DLB group was performed using Jaccard coefficient (J) for each module, with J = (number of shared regions within a module / total number of regions attributed to a module for both groups) x 100, expressed in %.

## Results

### Patients clinical characteristics

Both groups were comparable in terms of age and sex, whereas Controls had higher education attainment (mean ± SD 13.9 ± 2.8 vs. 12.1 ± 2.5 years, p = 0.03 uncorrected). As expected, MMSE and ACER scores were significantly lower in the DLB group (p < 0.001, *Mann-Whitney U test*) (Table 1). Prevalence of DLB diagnostic core features was as follows in our DLB group: 92% (22/24) had parkinsonism, 63% (15/24) had visual hallucinations and cognitive fluctuations, while 38% (9/24) had evidence of RBD. These results are in line with previous publications (Morbelli et al. 2019; Matar et al. 2020).

**Table 1:** Baseline characteristics of included subjects

	DLB (n = 24)	CTL (n = 23)	pval
Age *(years)*	74.3 ± 6.7 (62–87)	72.3 ± 5.7 (62–84)	0.27 *
Female proportion	16.7% (4/24)	34.8% (8/23)	0.15 $
Education *(years)*	12.1 ± 2.5 (8–17)	13.9 ± 2.8 (9–19)	0.03 #
MMSE	22.8 ± 4.3 (15–29)	28.9 ± 1.2 (26–30)	0.0001 #
ACER	67.7 ± 11.9 (45–87)	92.0 ± 6.6 (75–100)	0.0001 #
Mean cortical thickness	2.25 ± 0.13	2.37 ± 0.09	0.001 #

*t-test, $ Chi-Squared test, # Mann-Whitney U test

Seventeen DLB subjects had available ^11^C-Pittsburgh compound B (PiB) PET imaging, with 10 being amyloid-positive (cortical standardized uptake ratio (SUVR) > 1.5) and 7 amyloid-negative (cortical SUVR < 1.5).

### Global network analysis

**Fig. 1:**
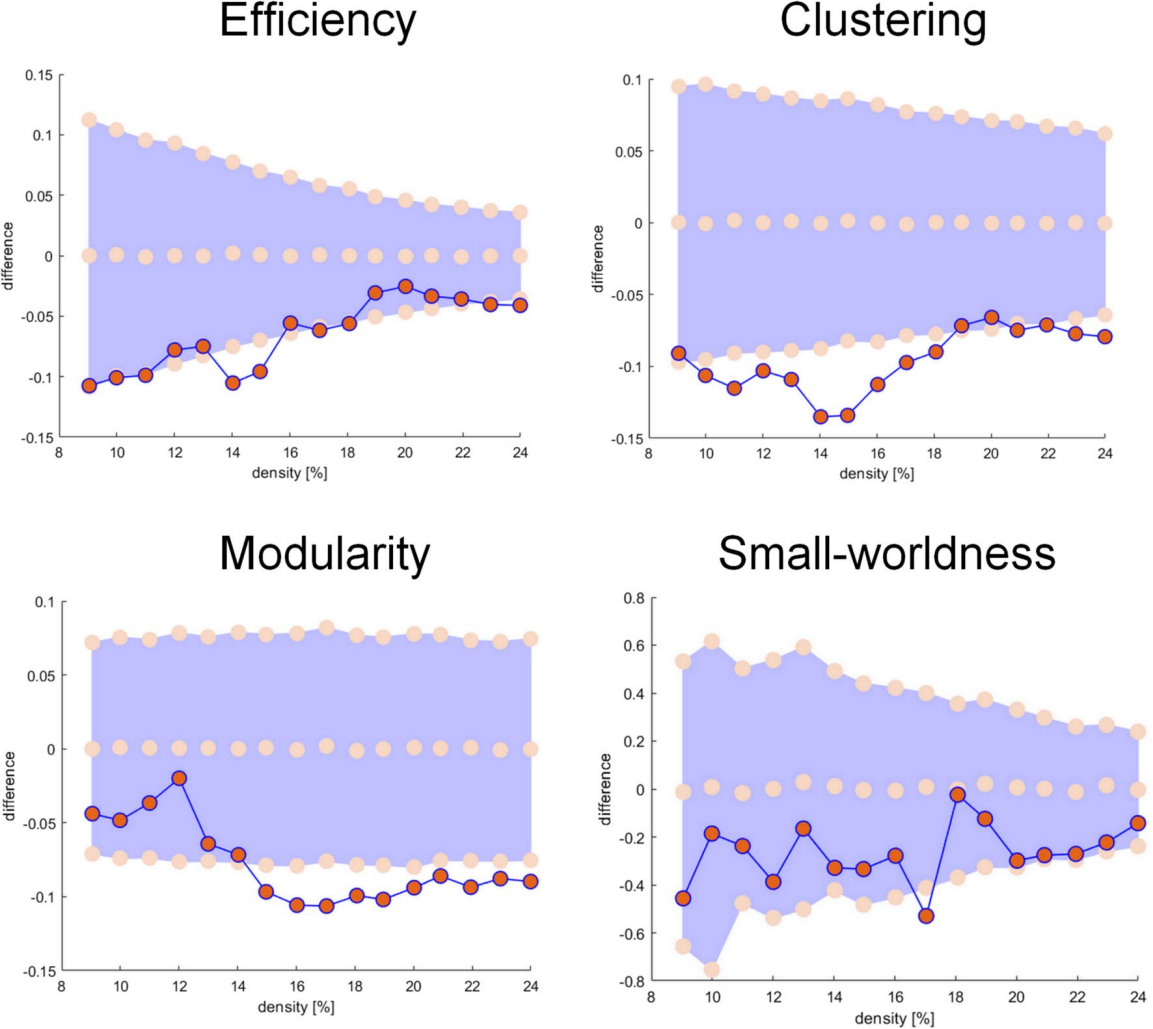
Differences between Controls and DLB participants regarding global measures assessed with densities between 9 and 24%. Negative difference indicates lower values for the DLB group (in red) compared to the Control group (pink dots with values centered on zero and 95% confidence interval represented in lavender)

Compared to Controls, DLB subjects showed significantly decreased local efficiency at D = 14–15% (p = 0.022), as well as decreased clustering coefficient (p = 0.043 − 0.006) and modularity (p = 0.044 − 0.020) at most D values. In addition, DLB had lower small-worldness (p = 0.002) at D = 17% (Fig. 1).

### Nodal network analysis

Compared to Controls, DLB subjects showed decreased nodal clustering in the right entorhinal and left middle temporal gyri (FDR q < 0.05) as well as decreased closeness centrality in extensive brain areas, including right rostral middle frontal, bilateral inferior frontal (pars opercularis), left paracentral, left isthmus and caudal anterior cingulate, left insula, right superior temporal, right fusiform, right transverse temporal, bilateral supramarginal, bilateral cuneus, left pericalcarine and right lingual gyri (FDR q < 0.05). We did not observe any significant differences regarding nodal efficiency.

### Modules

**Fig. 2:**
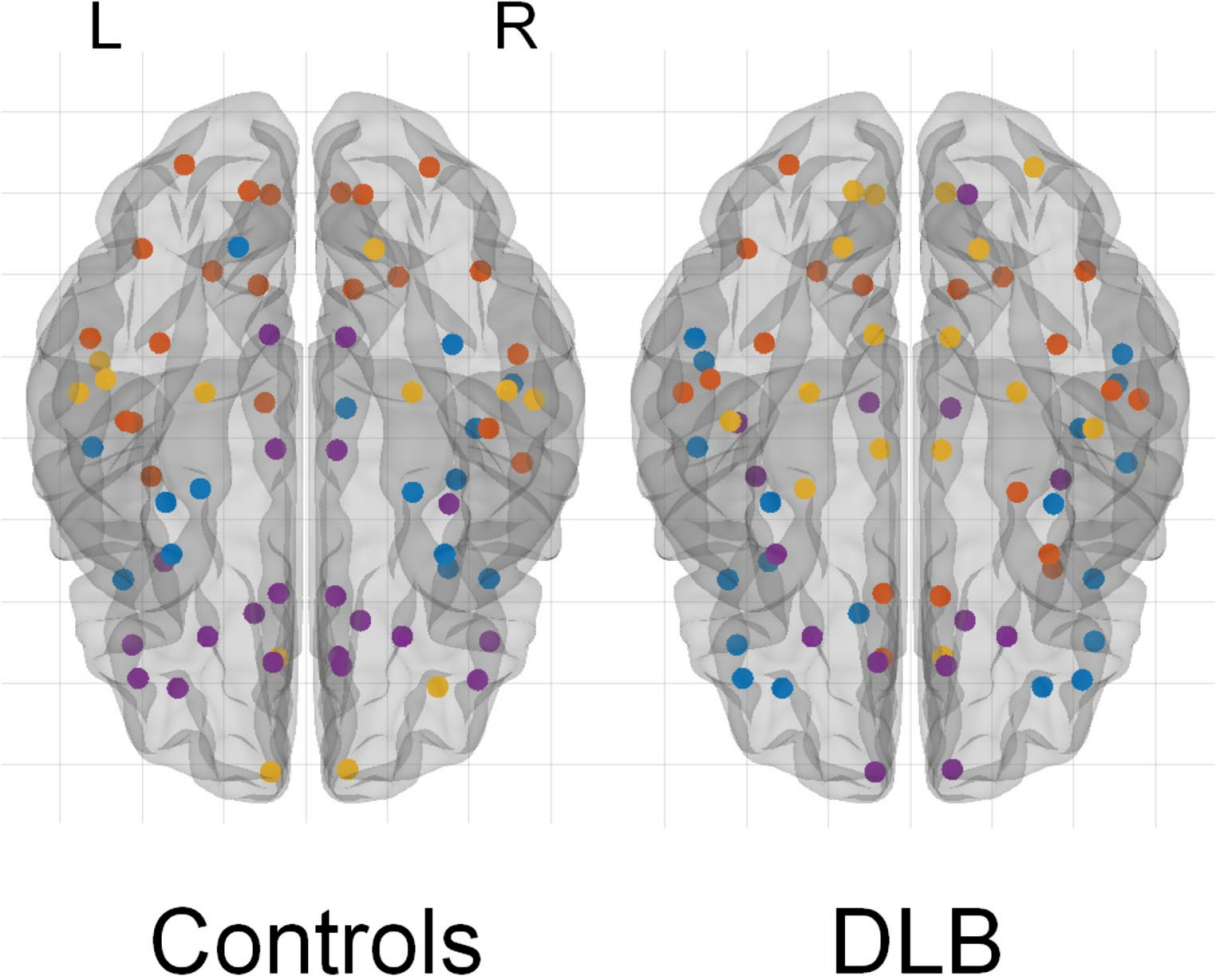
Brain modules in Controls and DLB participants. Both groups had four different modules. In Controls, Module I (blue) includes entorhinal, temporal pole and inferior frontal regions; Module II (orange) broadly mirrors dorsal attentional network, Module III (yellow) includes frontal pole and lateral temporal regions, while Module IV (violet) broadly corresponds to the default mode network. L and R indicate Left and Right

**Fig. 3:**
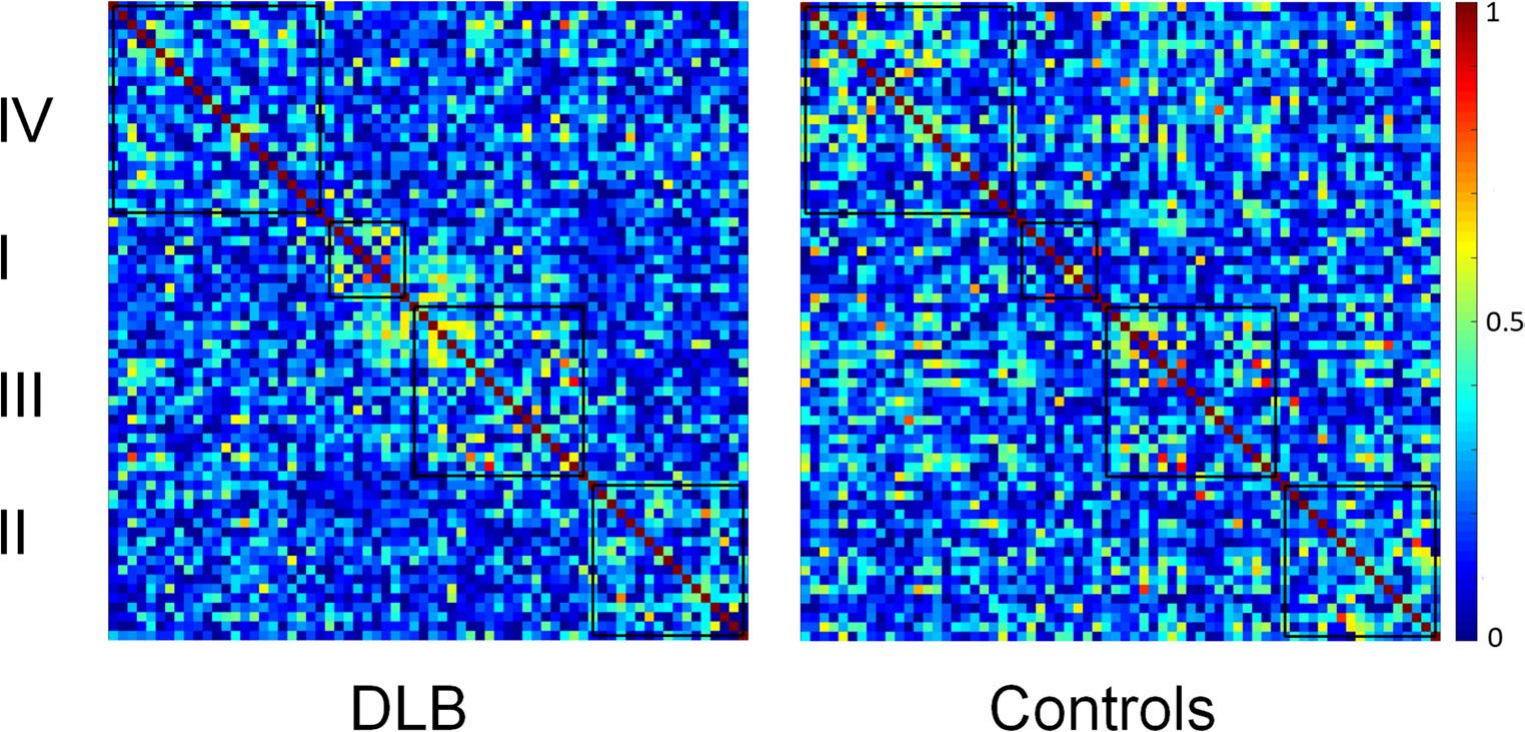
Weighted association matrix showing the four different modules (I-IV) in DLB and Controls. Color bar indicates the strength of the correlation coefficients (red/yellow represent stronger correlations while blue/green weaker correlations)

**Fig. 4:**
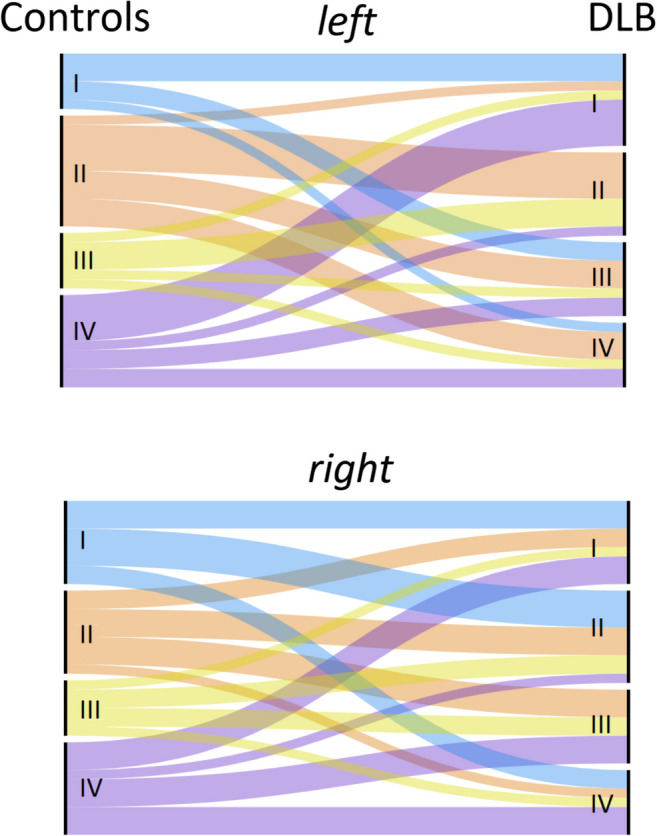
Alluvial plots showing the differential allocation of left and right hemispheric regions of interest into Modules for Controls (left) and DLB (right). See Table 2 for detailed regional differences

Four different modules were identified for Controls and DLB groups (Fig. 2). Regarding Controls, Module I (blue) included entorhinal gyrus, temporal pole and inferior frontal gyrus (pars opercularis). Module II (orange) encompassed precentral gyrus, parietal and occipital regions, broadly corresponding to the DAN (Vossel et al. 2014). Module III (yellow) included frontal pole and lateral temporal regions. Finally, Module IV (violet) included cingulate and orbitofrontal regions, partly mirroring DMN (Raichle 2015) (Fig. 2; Table 2). In comparison, we observed that DLB had disrupted networks, especially regarding inferior frontal, temporal, cingulate and occipital regions. For example, inferior frontal and cingulate areas were not part of Module IV (DMN), whereas pericalcarine gyrus and cuneus were not included into Module II (DAN), as well as weaker connectivity within modules, as shown in Fig. 3. A severe disruption of each module was observed for the DLB group, especially regarding Module III (J_III_ = 7.1%) and IV (J_IV_ = 14.7%), but also Module I (J_I_ = 17.6%) and Module II (J_II_ = 20%). Figure 4 shows the differential allocation of atlas regions to Modules I-IV for Controls and DLB.

**Table 2:** Brain modules in Controls and DLB patients. Roman numerals indicate the module assigned to each left and right ROI, respectively

Region of interest	Controls	DLB
Frontal lobe		
Superior frontal	IV / IV	I / IV
Rostral middle frontal	IV / III	I / I
Caudal middle frontal	IV / I	I / II
Inferior frontal (pars opercularis)	I / I	I / I
Inferior frontal (pars orbitalis)	IV / IV	I / I
Inferior frontal (pars triangularis)	IV / IV	I / I
Lateral orbitofrontal	IV / IV	IV / IV
Medial orbitofrontal	IV / IV	IV / IV
Frontal pole	III / III	IV / IV
Precentral	II / I	IV / IV
Paracentral	II / I	IV / IV
Temporal lobe		
Entorhinal	I / I	III / II
Parahippocampal	III / III	III / III
Insula	I / IV	I / I
Temporal pole	I / I	IV / II
Fusiform	II / I	II / II
Superior temporal	I / II	I / I
Middle temporal	III / III	II / II
Inferior temporal	III / III	II / II
Transverse temporal	II / II	III / III
Banks of superior temporal sulcus	II / II	I / I
Parietal lobe		
Postcentral	II / I	IV / I
Supramarginal	III / I	I / I
Superior parietal	II / II	II / II
Inferior parietal	II / II	II / II
Precuneus	II / II	II / II
Occipital lobe		
Lingual	I / III	III / III
Pericalcarine	II / II	III / IV
Cuneus	II / II	III / III
Lateral occipital	II / II	II / III
Cingulate cortex		
Rostral anterior	III / IV	II / III
Caudal anterior	IV / IV	II / II
Posterior	IV / IV	III / III
Isthmus	IV / IV	III / III

Roman numerals indicate the module assigned to each left and right ROI, respectively

## Discussion

This study confirms that DLB affects the graph theoretical properties of networks based on the covariance of cortical thickness. Specifically, the influence of DLB on global parameters (e.g. decreased efficiency, clustering, modularity, and small-worldness), indicates changes in large-scale structural connectivity networks. These data compare to the effect of AD, that decreases small-worldness and clustering (Phillips et al. 2015; Pereira et al. 2016), and Parkinson’s disease (PD) with MCI, that reduces global and nodal efficiency in frontal and parietal areas compared to Controls and PD without cognitive deficits. Together, these studies suggest that large-scale network disruptions are temporally related to the development of cognitive impairment (Pereira et al. 2015).

We found that DLB altered regional network topology, including decreased nodal clustering – a measure of segregation – and decreased closeness centrality. Clustering was reduced in temporal areas while closeness centrality was decreased in regions encompassing middle frontal gyrus, posterior cingulate and occipital regions. Previously, a reduced closeness centrality was observed for AD in temporoparietal regions (He et al. 2008; Yao et al. 2010). Using resting-state fMRI data, Schumacher et al. observed a decreased within-network connectivity in frontal and temporal areas in DLB (Schumacher et al. 2019). Previous studies have suggested that participation coefficient is preferred to centrality measures for correlational analyses (Power et al. 2013), but as we only performed group comparisons, we believe that closeness centrality is a suitable marker of nodal centrality. In addition, while participation can be considered as a centrality measure, it is rather a coefficient to estimate connections across different topological modules, with a high participation coefficient relating to a connector hub while a low participation coefficient suggests a provincial hub.

In addition to assessing global and regional topology measures, we computed large-scale network organisation into modules. Our results suggested that clearly delineated structural modules could be defined in Controls. These modules are reminiscent of functional brain networks described with fMRI, i.e. DMN including posterior cingulate and orbitofrontal regions and DAN including precentral and parietal areas (Vossel et al. 2014; Raichle 2015). Our DLB group showed extensive network disruptions (Fig. 4), with key regions being lost and assigned to other brain networks, especially inferior frontal, posterior cingulate and occipital cortex. These findings are in accordance with several fMRI studies showing disrupted DMN network in DLB (Chabran et al. 2018; Schumacher et al. 2019). Similarly, Sala et al. applied seed-based interregional correlation analyses using fluorodeoxyglucose PET, showing that DMN and DAN were particularly vulnerable large-scale networks in DLB (Sala et al. 2019).

As discussed by Phillips et al. (2015), the method used to construct the correlation matrix can dramatically affect not only the magnitude of the results, but also their direction. In fact, according to whether we use binary or weighted edges, Pearson or partial correlations, very different results have been observed even when using the same imaging modality. For example, characteristic path length has been shown to be either reduced (Tijms et al. 2013) or increased (He et al. 2008) in AD subjects compared to Controls. One explanation is that controlling for mean cortical thickness removes the effect of regional inter-dependencies. Thus, for graphs using Pearson correlations without controlling for mean cortical thickness, characteristic path length is usually lower in Controls than in groups affected by dementia, whereas for graph using mean cortical thickness as a covariate (as this was the case for the present study), the results tended to be in the opposite direction (Phillips et al. 2015). For this reason, we studied other global graph parameters (e.g. clustering coefficient) which do not show such discrepancies, in order to more easily compare our findings with the previous literature. Moreover, most studies used age and sex as covariates, but we believe that adjusting the data for education was critical as higher education attainment has been associated with larger cortical thickness (Querbes et al. 2009).

Our study has several limitations. First, our sample size was modest. However, we were able to include subjects from the same centre and using the same MRI scanner to reduce variance. In addition, our findings were subjected to a stringent significance threshold for regional graph parameters which allowed us to observe similarly significant findings than other multicentric studies with larger cohorts. Second, it would have been interesting to study the possibly differential pattern of large-scale network disruption in DLB harbouring an ADcopathology, as this affects around half of the patients. However, our small sample precluded such further analyses, as 17 of our 24 DLB subjects had an available PiB PET imaging (with 10/17 amyloid-positive subjects). Similarly, the small sample precluded further analyses correlating clinical core features (e.g. visual hallucinations or cognitive fluctuations) with global and nodal network alterations.

In summary, we present novel evidence of large-scale structural brain network impairment in DLB. Both global and local measures of efficiency and segregation are affected, offering new insights into the pathophysiology of neurodegeneration. Further studies including larger samples and prodromal DLB are required to confirm these findings and to tackle the complex relationship between structural and functional connectivity impairments in dementia.
